# No effect of autistic traits on social attention: evidence based on single-cue and conflicting-cues scenarios

**DOI:** 10.1186/s40359-024-01777-8

**Published:** 2024-05-27

**Authors:** Airui Chen, Meiyi Wang, Bo Dong

**Affiliations:** Department of Psychology, Suzhou University of Science and Technology, Xuefu Road 99, Suzhou, 215009 China

**Keywords:** Social attention, Autistic traits, Cue-target task, Single-cue, Conflict-cue scenario

## Abstract

Individuals often use others’ gaze and head directions to direct their attention. To investigate the influence of autistic traits on social attention, we conducted two experiments comparing groups with high and low autistic traits in single-cue (Experiment 1) and conflicting-cue (Experiment 2) scenarios. Our findings indicate that individuals responded more rapidly to the direction of a single social cue or the consensus of multiple cues. However, we did not observe significant differences in social attention between individuals with high and low autistic traits. Notably, as the stimulus onset asynchrony (SOA) increased, individuals with low autistic traits exhibited greater improvements in reaction speed compared to those with high autistic traits. This suggests that individuals with low autistic traits excel at leveraging temporal information to optimize their behavioral readiness over time, hinting at potential variations in cognitive flexibility related to autistic traits.

## Introduction

Interpersonal communication problems among college students are adversely affecting their mental health. During interpersonal interaction, crucial information is conveyed through various non-verbal cues such as the direction of human eye gaze, body orientation, and head orientation. These cues encompass a wealth of information, including the focus of attention, inner thoughts, intentions, and the goal of the action [1–8]. Observers tend to follow other’s cue direction to redirect their attention, referred to as social attention [2, 5, 9–15]. Accurately perceiving cues and social attention plays a vital role in fostering positive interpersonal relationships [6, 16–18]; conversely, communication and interaction difficulties may arise when there are deficits in processing social cues and social attention. A typical case is evident within the autism symptoms, where individuals with autism exhibit significant challenges, including a diminished capacity for establishing eye contact, an inability to follow another person’s gaze, and difficulty in maintaining shared attention on the same object with others [19–21].

Similarly, individuals with high autistic traits in typically developing adults, not meeting clinical criteria for an autism diagnosis, exhibit weaker social communication abilities and are at an increased risk for developing interpersonal communication disorders [22, 23]. It has been found that individuals with high autistic traits differ from normal individuals in attentional orienting, i.e., focusing more on the details of things than on the whole, which is one of the typical performances of individuals with autism [24–26]. Based on the findings of autism research, individuals with high autistic traits may have deficits in their ability of social attention, which is essential for interpersonal communication.

However, it is not clear whether individuals with high autistic traits have lower abilities in social attention, as existing research exhibits divergence. While specific studies indicate that individual differences associated with autistic traits influence social attention [27–29], others do not support this viewpoint [25, 38]. In studies focusing on gaze perception, it was discovered that observing gaze cues, particularly during social interactions and communications, alters our subjective experience of time, making it appear to pass faster than it actually does. Importantly, the strength of this compression effect negatively correlates with scores of autistic traits [24, 25]. The research findings on whether autistic traits affect the perception of social gaze cues and social attention are inconsistent. These discrepancies may stem from variations in task scenarios and experimental materials (e.g., real faces or schematic faces). These inconsistencies prompt further exploration into whether autistic traits indeed affect social attention. To more thoroughly investigate this issue and minimize the potential impact of facial stimuli on the main outcomes, it’s best to include both real and schematic faces within the same experiment for more reliable results.

In classic social attention studies, researchers usually use the Posner cuing paradigm to investigate social attention [13, 30–36]. Typically, a face with a left or right gaze is presented in the center of the screen, with the subsequent appearance of a target appearing on the left or right as either a valid or invalid cue condition. Participants exhibit faster responses in the valid cue condition compared to the invalid cue condition, indicating their ability to engage in social attention. However, it’s noteworthy that the gaze cue in these studies often involves a single model rather than a group of models. In real-life situations, individuals frequently engage with a crowd of people, each presenting conflicting social cues. In such social scenarios, individuals tend to follow the social cues of the majority to shift their attention [31]. Nevertheless, it remains unclear whether individuals with high autistic traits would similarly follow the social cues of the majority.

To probe these issues, we investigated individual differences in social attention related to autistic traits within two scenarios: a single social cue (experiment 1) and multiple conflicting cues (experiment 2). We used the Posner cueing paradigm to measure attentional effects as the indicator of social attention, as in previous studies [33]. Comparing the attentional effects between individuals with low and high autistic traits would help us to elucidate whether individual differences related to autistic traits manifest in social attention.

## Experiment 1

To explore the impact of autistic traits on social attention in a single gaze scenario, we utilized the Posner cueing paradigm, presenting both real and schematic faces. This allowed for a comparison of attentional effects between a group with high autistic traits and a group with low autistic traits.

### Methods

#### Participants

G*Power 3.1.9.7 [32] was used to estimate the sample size for a 2 × 2 × 2 × 2 four-way repeated measures analysis of variance (ANOVA) (f = 0.25, alpha = 0.05, power = 0.95), yielding that a sample size of *N* = 24 for each group was sufficient to achieve the desired statistical power. The Chinese version of the Autism-Spectrum Quotient Questionnaire [19, 26] was employed to assess participants’ autistic traits, with higher scores indicating elevated levels of autistic traits. Initially, 445 valid AQ questionnaires were randomly distributed among college students [37]. Participants with the top 10% scores were selected for the high autistic trait group, while those with the bottom 10% were chosen for the low autistic trait group. For detailed statistical information regarding the 445 data, refer to [37]. We arranged the 445 questionnaires in order according to the scores from highest to lowest. These 90 students were then invited to partake in our experimental study. Considering their consent to participate and our required sample size, 60 students (17 males and 43 females) from the aforementioned group of 90 students took part in Experiment 1. These individuals were aged between 18 and 23 years and included an equal split of 30 from the top 10% and 30 from the bottom 10%. All participants provided written informed consent. The range of the high AQ scores was 130–150, and the range of the low AQ scores was 80–103. Notably, we performed the t test and a significant difference in AQ scores was observed between the high AQ group (*M* = 134.30, *SD* = 3.91) and the low AQ group (*M* = 96.73, *SD* = 4.34), *t*(58) = 35.21, *p* = 0.001. The study received approval from the ethics committee at Suzhou University of Science and Technology. The approval date is 2020.03.14 and the approval number is N.202,031,475. And at the end of the experiment, we paid 20¥ to each participant.

#### Apparatus, stimuli, and experimental setup

The experimental program was coded in Eprime 2.0 and executed on a computer with a resolution of 1920 × 1080 at 100 Hz. Participants responded using an IBM keyboard. The visual display featured a gray background (RGB: 128, 128, 128), a central black “+” as the focus point and a black asterisk “*” (1.5°) as the target. The distance between the central focus point and the target was 7°, and the size of the central focus point was set to be 1.5 times the size of the target. The stimuli consisted of two types of faces: real faces and schematic faces, depicting either a left or right gaze. In this study, the photos of real faces displaying neutral emotions were chosen from the Chinese Facial Affective Picture System (CFAPS) [38].

#### Experimental design and procedure

**Fig. 1:**
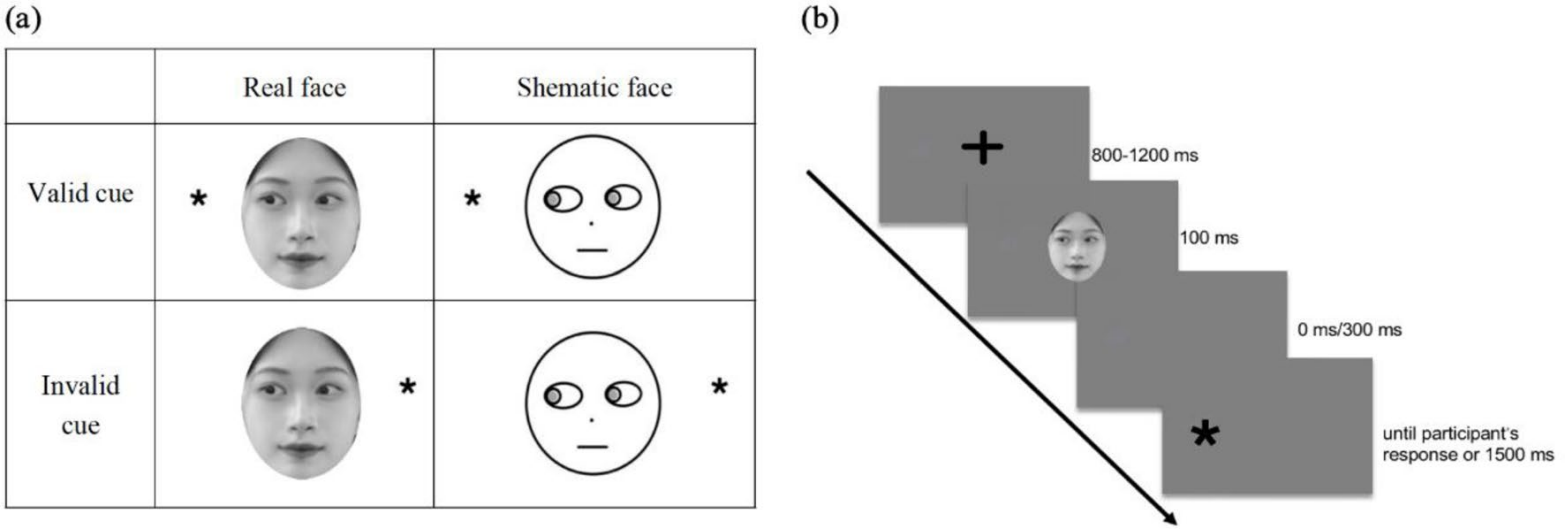
The conditions (**a**) and experimental procedure in a trial (**b**)

The experimental procedure for a single trial is illustrated in Fig. 1. We employed a 2 × 2 × 2 × 2 mixed design, incorporating cue validity (valid cue vs. invalid cue), face type (real face vs. schematic face), and Stimulus Onset Asynchrony (SOA: 100 ms vs. 400 ms) as within-participant variables. Autistic traits (High AQ vs. Low AQ) were introduced as a between-participant variable.

**Fig. 2:**
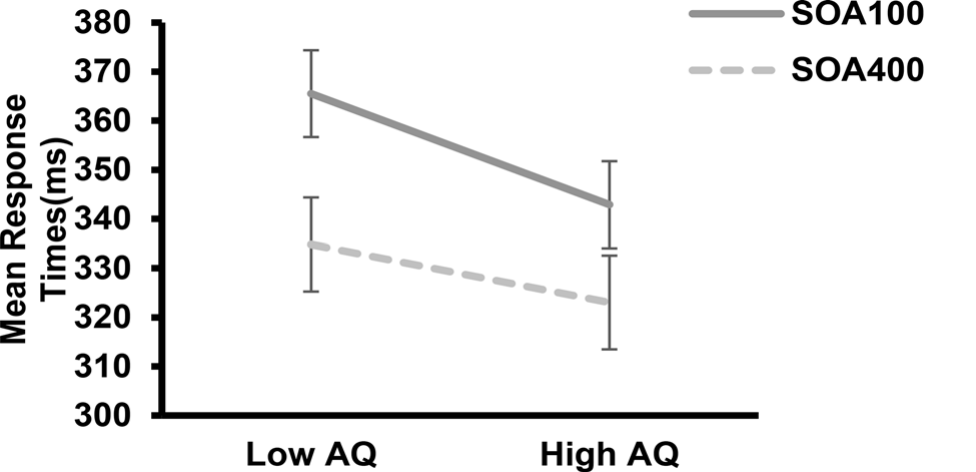
Mean response times under different SOAs conditions for real face and schematic face. Error bars represent standard errors of the mean

The experiment was conducted in a dimly lit room, and participants were positioned 70 cm away from the screen with their heads resting on a chin rest. The experimental procedure is depicted in Fig. 2. Initially, a central gaze cue was displayed in the center of the screen for a duration of 800–1200 ms. Subsequently, a face with either left or right gaze was randomly presented on the left or right side for 100 ms, followed by an empty screen randomly presented for either 0 ms or 300 ms. Finally, the target “*” was randomly presented on the left or right side of the screen, with a maximum presentation time of 1500 ms. Participants were instructed to respond promptly and accurately by pressing the left arrow key for the left location and the right arrow key for the right location. Once participants pressed the key, the target disappeared. Before the formal test, participants underwent a practice session with 10 trials. The accuracy of all the participants in the practice task was higher than 80%, indicating they understand the task. The formal experiment comprised 288 trials distributed across four blocks, with 72 trials in each block. Rest periods were provided between each pair of consecutive blocks. Within each block, only one type of face was presented, and the order of face-type presentation was counterbalanced across participants. For half of the participants, gaze cues were presented on schematic faces in the first two blocks and on real faces in the last two blocks, while the order was reversed for the other half of the participants.

### Results

In Experiment 1, all the participants completed the experiments carefully, with a mean accuracy of *M* = 99.5%, *SD* = 0.048. Table 1 displays the mean accuracy and reaction times (RTs) under each condition.

**Table 1:** Mean of average RTs on correct trials and mean accuracy (with standard errors in parentheses) for each condition

			cue validity			
			valid cue		invalid cue	
			RTs	accuracy	RTs	accuracy
Low AQ	100ms	Schematic face	358.18(10.39)	99.7(0.20)	365.11(9.96)	99.8(0.20)
		Real face	366.08(9.67)	99.6(0.20)	372.68(9.69)	99.5(0.30)
	400ms	Schematic face	329.54(9.53)	99.6(0.20)	337.29(10.14)	99.4(0.20)
		Real face	332.37(8.67)	99.8(0.20)	340.03(8.72)	99.5(0.20)
High AQ	100ms	Schematic face	338.40(10.40)	99.4(0.20)	345.83(9.56)	99.1(0.20)
		Real face	340.78(9.57)	99.6(0.20)	346.56(9.70)	99.3(0.30)
	400ms	Schematic face	321.67(9.53)	99.7(0.20)	330.09(10.14)	99.5(0.20)
		Real face	318.48(8.67)	99.6(0.20)	321.86(8.72)	99.6(0.20)

*Note* with standard errors in parentheses

A 2(valid cue vs. invalid cue) × 2 (autistic traits: High AQ vs. Low AQ) × 2 (face type: real face vs. schematic face) × 2 (SOA: 100 ms vs. 400 ms) mixed ANOVA was conducted for RTs. The results revealed a significant main effect of cue validity, *F*(1, 58) = 53.79, *p* = 0.001, *η*_*p*_^*2*^ = 0.48, 95%CI = [-8.58, -4.90], indicating significantly faster responses in the valid cue condition than in the invalid cue condition. Additionally, a significant main effect of SOA was observed, *F*(1, 58) = 162.52, *p* = 0.001, *η*_*p*_^*2*^ = 0.74, 95%CI = [21.31, 29.25], showing quicker responses at SOA_400_ compared to SOA_100_. This outcome demonstrates the foreperiod effect, where the shorter duration between a warning signal and the subsequent stimulus leads to faster response times [39, 40]. There was no significant main effects of face type (*F*(1,58) = 0.223, *p* = 0.638, *η*_*p*_^*2*^ = 0.04) and autistic traits (*F*(1, 58) = 1.786, *p* = 0.187, *η*_*p*_^*2*^ = 0.30).

**Fig. 3:**
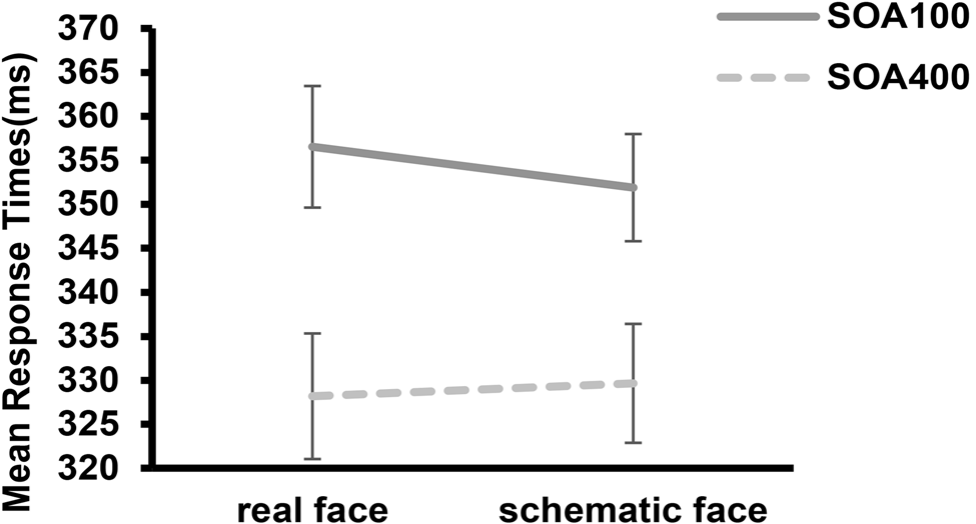
Mean response times under different SOAs conditions for low AQ group and high AQ group. Error bars represent standard errors of the mean

Importantly, there was a significant interaction between different autistic traits and SOA (*F*(1, 58) = 7.46, *p* = 0.008, *η*_*p*_^*2*^ = 0.114). Further analysis for the interaction of autistic traits and SOA, we calculated the foreperiod effect by subtracting RTs under SOA_100_ and SOA_400_ for high AQ and low AQ individuals respectively, revealing a larger foreperiod effect observed in low AQ (*M* = 30.70 *SD* = 15.75) compared to high AQ (*M* = 19.87, *SD* = 14.96), *t*(58) = 2.73, *p* = 0.008, see Fig. 3.

Furthermore, the results revealed a significant interaction between face type and SOA (*F*(1, 58) = 7.435, *p* = 0.008, *η*_*p*_^*2*^ = 0.114). Subsequent analysis demonstrated that the foreperiod effect for real faces (*M* = 28.34, *SD* = 16.96) was larger than that for schematic faces (*M* = 22.23, *SD* = 19.61), *t*(59) = 2.744, *p* = 0.008, see Fig. 2. The other interactions were not significant, *p* > 0.05.

## Experiment 2

### Methods

#### Participants

31 participants in experiment 1 were willing to participate in experiment 2 and they participated in experiment 2 one month after participating experiment (1) Another 29 participants only participate in experiment (2) Therefore, 60 participants (11 males and 49 females) were recruited. A significant difference in Autism-Spectrum Quotient (AQ) scores was observed between the high AQ group (*M* = 134.3, *SD* = 4.55) and the low AQ group (*M* = 96.23, *SD* = 4.89), *t*(58) = 31.16, *p* = 0.001. All participants had normal visual acuity or corrected visual acuity, exhibited no color blindness or color deficiency, and were right-handed. The study received approval from the ethics committee at Suzhou University of Science and Technology. The approval date is 2020.03.14 and the approval number is N.202,031,475. And at the end of the experiment, we paid 20¥ to each participant.

#### Apparatus, stimuli, and experimental setup

The experimental program for Experiment 2 was executed on a computer with a resolution of 2560 × 1440 at 100 Hz. The visual display featured a gray background, with a black “+” serving as the central gaze point and the black letter “T” as the target.

Based on the materials from Sun et al. [41], we also created the virtual characters, that is, avatars. A total of 10 avatars, consisting of 6 males and 4 females, were chosen to simulate natural social groups in Experiment 2. Each character had three types of head orientation: the first one facing forward (all characters appeared in the center of the screen, looking directly at the subjects); the second one tilting the head 30° to the left; and the third one tilting the head 30° to the right. The direction of gaze remained constant throughout the experiment with the same head orientation, and all avatars displayed neutral facial expressions (refer to the Nimstim Expression Gallery for facial expressions [42]). All stimuli were presented on a gray background (RGB: 80, 80, 80). The experiment employed the conflict scenario from the research by Sun et al. [41], with a majority number of 9 and a minority number of 1. The probability of each character becoming the minority was equal.

The 3D animation software Poser 6® (E Frontier, Scotts Valley, California, USA) was employed to create six different arrangements of 10 avatars, resulting in distinct scenarios for each character’s appearance. To simulate a realistic social group scenario, the ten characters were organized into three rows, with a consistent probability assigned to each character’s appearance in each row. To enhance visual proximity, the characters were configured as follows: three characters in the first row (visual angle: 4° × 8°; head size: 1.5° × 2°), three characters in the second row (visual angle: 3.5° × 7°; head size: 1° × 1.5°), and four characters in the third row (visual angle: 3° × 6°; head size: 1° × 1.5°). The visual angle of the entire group photo was set to 12° × 17°. The visual angle of the target (the letter T) that participants needed to judge the location was established at 0.4° × 0.4°, with an eccentricity of 13°.

#### Experimental procedures and design

**Fig. 4:**
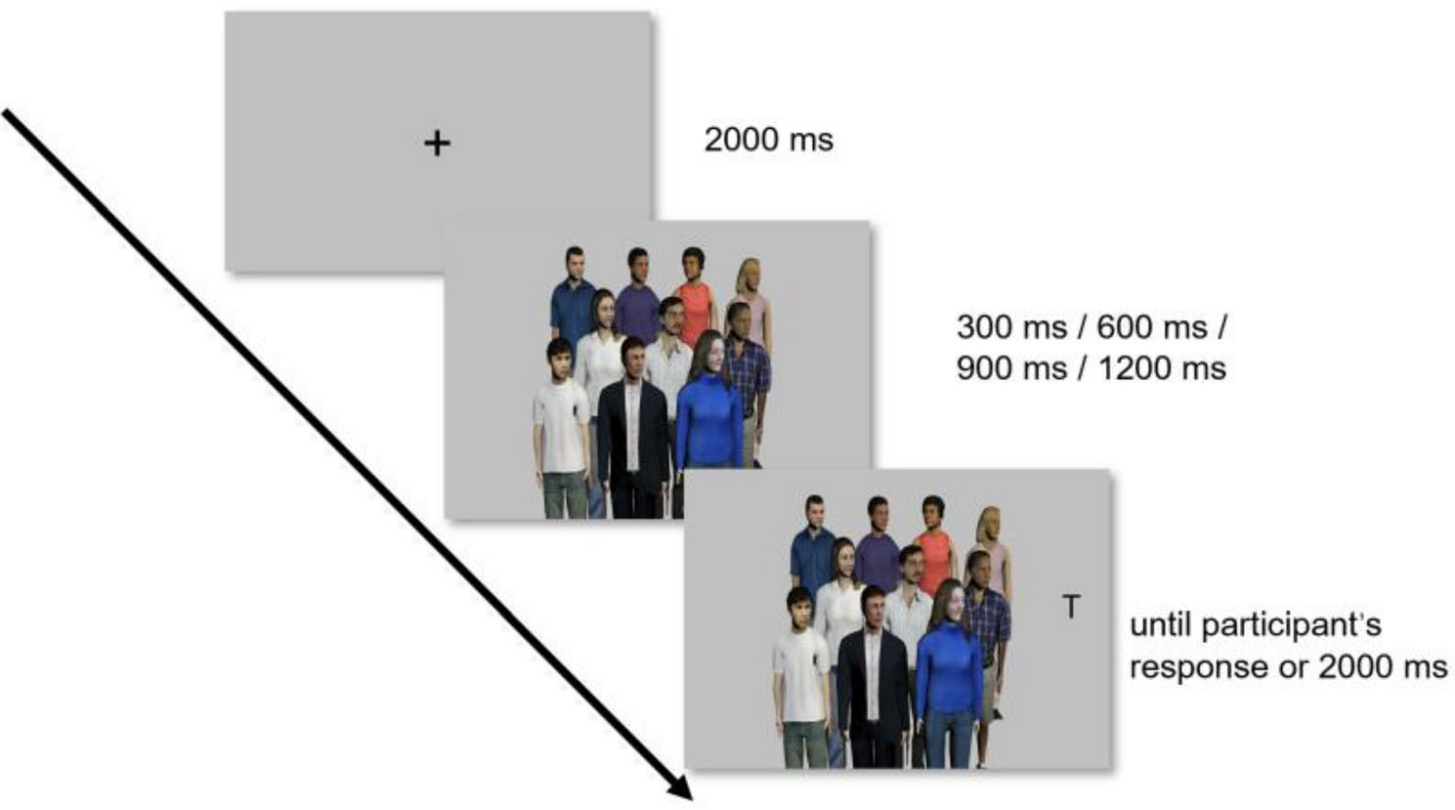
The experimental procedure in a trial

The experiment followed a 2 × 2 × 4 mixed design, incorporating cue validity (valid cue vs. invalid cue) and SOA (300 ms vs. 600 ms vs. 900 ms vs. 1200 ms) as within-participant variables, and autistic traits (High AQ vs. Low AQ) as between-participant variable. The experiment was conducted in a dimly lit room, with participants seated in front of the screen at a distance of 70 cm, and their heads rested on a chin rest. The procedural details are illustrated in Fig. 4. Initially, a central fixation point was presented in the center of the screen for 2000 ms. Subsequently, the multiple gaze photo was displayed for 300 ms, 600 ms, 900 ms, or 1200 ms. Finally, the target “T” was randomly presented on the left or right side of the screen with a maximum presentation time of 2000 ms. Participants were tasked with providing quick and accurate responses, pressing the “F” key if the “T” appeared on the left and the “J” key if the “T” appeared on the right. The experiment comprised 480 trials distributed across eight blocks, with 60 trials in each block.

### Results

In Experiment 2, all the participants completed the experiments carefully, with a mean accuracy of *M* = 99.5%, *SD* = 0.012. Table 2 displays the mean accuracy and reaction times (RTs) under each condition.

**Table 2:** Mean of average RTs on correct trials and mean accuracy (with standard errors in parentheses) for each condition

		Consistency of target location and cue validity			
		valid cue		invalid cue	
		RTs	accuracy	RTs	accuracy
High AQ	300ms	395.95(8.94)	99.7(0.20)	386.57(8.93)	99.73(0.20)
Low AQ		403.55(8.94)	99.4(0.20)	394.68(8.93)	99.67(0.20)
High AQ	600ms	370.97(7.81)	99.8(0.20)	361.12(7.85)	99.80(0.20)
Low AQ		383.49(7.81)	99.2(0.20)	373.66(7.85)	99.30(0.20)
High AQ	900ms	363.57(7.84)	99.7(0.20)	348.57(6.88)	99.63(0.40)
Low AQ		371.91(7.85)	99.4(0.20)	356.36(6.88)	99.53(0.40)
High AQ	1200ms	357.19(7.40)	99.9(0.40)	352.12(7.58)	99.85(0.20)
Low AQ		368.02(7.40)	99.2(0.40)	358.56(7.58)	99.63(0.20)

*Note* with standard errors in parentheses

**Fig. 5:**
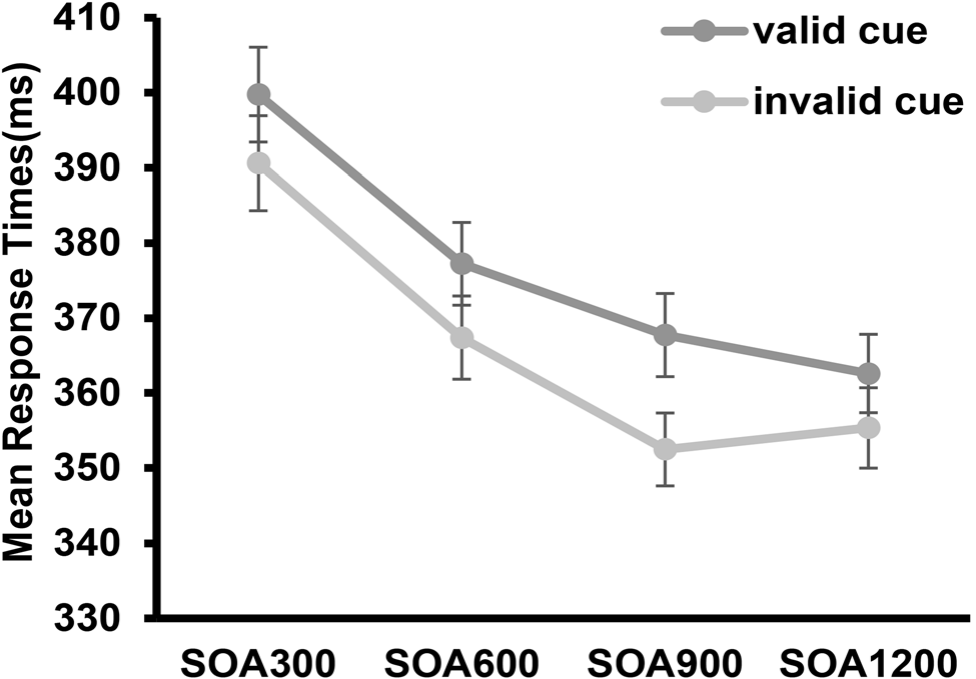
Mean response times for each condition. Error bars represent standard errors of the mean

A 2(cue validity: cued vs. uncued) × 2 (autistic traits: High AQ vs. Low AQ) × 4 (SOA: 300 ms vs. 600 ms vs. 900 ms vs. 1200 ms) mixed ANOVA was conducted for RTs. Significant main effects were observed for cue validity, *F*(1,58) = 94.76, *p* = 0.001, *η*_*p*_^*2*^ = 0.62, 95%CI = [8.24, 12.51], with participants responding significantly slower in the invalid cue condition than in the valid cue condition, and SOA, *F*(3, 56) = 62.93, *p* = 0.00,*η*_*p*_^*2*^ = 0.771, 95%CI = [1.502, 41.651], indicating that SOA300 RTs < SOA600 RTs < SOA900 RTs < SOA1200 RTs. There was no significant main effect of autistic traits (*F*(1, 58) = 0.746, *p* = 0.391, *η*_*p*_^*2*^ = 0.013). Additionally, there was a significant interaction between cue validity and SOA (*F*(3, 56) = 2.772, *p* = 0.05, *η*_*p*_^*2*^ = 0.13), see Fig. 5. Further analysis was conducted for cue validity and SOA, which revealed that the amount of attentional effect for SOA_300_ (*M* = 9.13, *SD* = 2.16) was smaller than the amount of attentional effect for SOA_900_ (*M* = 15.27, *SD* = 2.11), *p* = 0.037; the amount of attentional cue effect for SOA_600_ (*M* = 9.44, *SD* = 1.81) was smaller than the amount for SOA_900_, *p* = 0.046; the amount of attentional cue effect for SOA_1200_ (*M* = 7.01, *SD* = 2.00) was smaller than the amount for SOA_900_*p* = 0.05. No significant interaction differences were found in all other conditions, *p* > 0.05.

## Discussion

To explore the impact of autistic traits on social attention, we compared groups with high and low autistic traits in both a single social cue (Experiment 1) and conflicting cues (Experiment 2) scenarios. Our findings revealed that individuals responded more rapidly to the direction of a single social cue or the majority of multiple cues than if the target appeared in the opposite direction, which is consistent with previous research [10–13, 41]. More importantly, no discernible differences in social attention were identified between individuals with high and low autistic traits, regardless of whether the experimental materials consist of a schematic face, a real face, or faces of multiple people. In both experiments, we consistently observed a significant foreperiod effect, indicating that reaction speeds accelerated as the Stimulus Onset Asynchrony (SOA) intervals increased. Notably, this effect was more pronounced in individuals with lower autism trait levels compared to those with higher levels, but this was only evident when participants viewing a single face. In terms of experimental materials, real faces showed a more substantial foreperiod effect than schematic faces at varying SOA intervals.

In the two experiments, we did not observe differences in the gaze cue effect between individuals with high and low autistic traits. Specifically, individuals with high autistic traits exhibited the capacity to use individual gaze cues to orient their attention, and they were also proficient in following majority social gaze cues in conflicting social scenarios. Despite our discrepancy with certain research findings [27–29], similar findings were reported in another single cue task, where individuals with high and low autistic traits demonstrated comparable abilities in social attention [43, 44]. The absence of differences in gaze cue effects between individuals with high and low autistic traits may stem from the possibility that those with high autistic traits employ compensatory non-social strategies during the gaze-cueing task. For instance, individuals with high autistic traits may interpret the direction of the social cue as a non-social cue, such as an arrow. Consequently, in straightforward tasks that lack high social functionality, individuals with high autistic traits may exhibit similar social attention abilities as those with low autistic traits. Furthermore, previous studies revealed a strong negative correlation between autistic traits and phenomena such as gaze-induced time distortion and time compression [43, 45]. This evidence suggests that differences associated with autistic traits may manifest more prominently in tasks requiring higher social-cognitive capabilities. For instance, mental states [46] and the context of face perception [47] may play mediate role in the relationship between autist traits and social attention. Future investigations should explore whether autistic traits influence gaze perception and related behavioral performances, particularly when participants must process richer social information to finish social cue-related tasks.

Consistent with earlier research on social attention [31], our study revealed a foreperiod effect, indicating that the longer the interval between the cue and the target, the faster participants responded. Although the cue did not assist participants in judging the target’s location, it served as an event cue, suggesting the imminent presence of a target that requires judgment and leads to faster responses. This foreperiod effect has been explained as a change in the endogenous preparation state over time, reflecting a fundamental cognitive capacity within the human cognitive system [48, 49]. Interestingly, although we did not find differences in social attention abilities between individuals with high and low autistic traits in Experiment 1, we observed that individual differences related to autistic traits do manifest in the foreperiod effect. Compared to those with high autistic traits, individuals with low autistic traits exhibited a larger foreperiod effect as Stimulus Onset Asynchrony (SOA) increased, indicating that the enhancement in reaction speed for individuals with low autistic traits surpassed those with high autistic traits. Participants exhibiting lower levels of autistic traits demonstrate enhanced utilization of temporal information to optimize their behavioral preparation over time. This observation implies that, in contrast to individuals with higher autistic traits, those with lower autistic traits exhibit greater cognitive adaptability. In alignment with our findings, individuals characterized by lower autistic traits exhibit heightened levels of scientific reasoning abilities and reduced inclination towards conspiracy theory beliefs [50]. Conversely, older adults demonstrating higher autistic traits encounter increased challenges when engaging in tasks that assess working memory and sustained attention [51]. These findings imply the existence of potential variations in cognitive flexibility related to autistic traits. In future research, it would be valuable to explore whether these individual differences in cognitive flexibility manifest at the perceptual or motor response level.

Compared to schematic, we observed a more pronounced foreperiod effect for real faces. Specifically, our brain processes the real faces differently from schematic faces, particularly in the facial expression [52, 53] and emotion detection [54]. Thus, our finding may be attributed to the fact that real faces convey a richer array of social information, such as gender, age, nationality, etc., re more realistic, potentially leading to a distinct temporal course compared to that of schematic faces. In future investigations pertaining to social cues, it is imperative to contemplate the distinction between rudimentary geometric representations, such schematic faces, and authentic facial depictions within the context of experimental materials. Furthermore, there is a compelling rationale for the incorporation of socio-cognitive stimuli that better align with real-world ecological social cues.

## Conclusions

In summary, the present study provides clear evidence that individuals with both high and low autistic traits adeptly utilize single gaze cues and the majority of conflicting social cues to direct attention. Notably, our findings highlight a more pronounced foreperiod effect for individuals with low autistic traits, indicating their better ability to leverage temporal information for optimizing behavioral preparation over time. Further investigation into the processing of social cues and their influence on autism traits across diverse tasks and social contexts has the potential to advance the development of treatments for individuals with autism.

## Data Availability

The data used in the present study are available from the corresponding author upon reasonable request.
